# Mycetoma in the Togolese: An Update from a Single-Center Experience

**DOI:** 10.1007/s11046-018-0260-y

**Published:** 2018-03-20

**Authors:** Tchin Darré, Bayaki Saka, Abas Mouhari-Toure, Mazamaesso Tchaou, Améyo Monique Dorkenoo, Kwamé Doh, Atchi Walla, Koffi Amégbor, Vincent Palokinam Pitché, Gado Napo-Koura

**Affiliations:** 1 Department of Pathology, University Teaching Hospital of Lomé, Lomé, Togo; 2 Department of Dermatology, University Teaching Hospital of Lomé, Lomé, Togo; 3 Department of Radiology, University Teaching Hospital of Lomé, Lomé, Togo; 4 Department of Parasitology and Mycology, University Teaching Hospital of Lomé, Lomé, Togo; 5 Department of Trauma, University Teaching Hospital of Lomé, Lomé, Togo; 6 University of Lomé, BP 1515, Lomé, Togo

**Keywords:** Mycetomas, Epidemiology, Etiological agents, Togo

## Abstract

**Background:**

Mycetoma is a chronic inflammatory process caused either by fungi (eumycetoma) or bacteria (actinomycetoma). In this retrospective study, we report epidemiologic and histopathological data of mycetoma observed in the Lome Hospital, Togo in a 25-year period (1992–2016).

**Methodology:**

This is a retrospective study, over a period of 25 years, to analyze epidemiological and etiological findings of mycetomas seen in the single laboratory of pathological anatomy of the Lomé, Togo.

**Results:**

A total of 61 cases were retrieved from which only 33 cases were included which where clinically and microbiologically confirmed. The mean age of the patients was 29.7 ± 1.34 and a sex ratio (M/F) of 1.5. The majority of patients were farmers (*n* = 23 cases; 69.7%). Diagnosed etiologic agents were fungal in 24 cases (72.7%) and actinomycotic cases in 9 cases (27.3%). The fungal mycetomas consisted of *Madurella mycetomatis* (black grains) and *Falcifomispora senegaliensis* (black grains). The actinomycotic agents were represented by *Actinomadura madurae* (white grains), *Actinomadurae pelletieri* (red grains) and *Nocardia* sp. (yellow grains).

**Conclusion:**

This report represents a single-center study which provides epidemiologic and histopathological data of mycetoma cases in Togo.

## Introduction

Mycetoma is pathological processes in which fungal or actinomycotic agents of exogenous origin produce grains [1]. They are due to the inoculation of these pathogens during a trauma by a spine contaminated in semi-desert zone [1, 2]. It is a chronic infectious disease of soft tissue and bones with sometimes fatal visceral attacks [3]. The mycetoma endemic zone is located in the northern hemisphere on both sides of the 15th parallel [3]. This zone of predilection mainly includes Mexico, Sahelian Africa and India [4].

The West African endemic area, defined by annual rainfall ranging from 100 to 800 mm, includes Mali, Mauritania, Niger and Senegal [5]. In this area, mycetomas represent a public health problem [5, 6]. Their epidemiological, clinical and etiological aspects have been studied in different countries [2, 3, 6, 7]. A single publication was made on these mycetomas in Togo which could be considered “geographically spared” by this mycosis [8], because Togo lies entirely outside this rainfall zone. This work gathers the cases of mycetomas diagnosed in the laboratory of pathological anatomy of Lomé. It aims to clarify the epidemiological and etiological aspects of this disease.

## Methodology

This is a retrospective (cross-sectional) analysis of the database, and histopathological records of the Department of anatomical pathology at the General Hospital of Lome, patients were enrolled between 1992 and 2016 (25 years). We included all cases of mycetoma confirmed by histopathological observation of grains using a variety of histochemical stains like hematoxylin and eoisn (H and E), periodic acid Sciff’s (PAS) and Grocott’s methenamine silver (GMS) [9, 10]. The parameters studied were epidemiological (age, sex, geographical origin, occupation) and diagnosis (lesion site, lesion description, nature of biopsy, laboratory diagnosis).

### Ethical Clearance

This study received approval from the head of the laboratory department to be conducted (Ref No. 08/2017/LAP/CHUSO). Since it was counting records, patient consent was not required. However, during the counting and data collection patient names were not collected in order to preserve confidentiality.

## Results

### Epidemiological Data

**Fig. 1:**
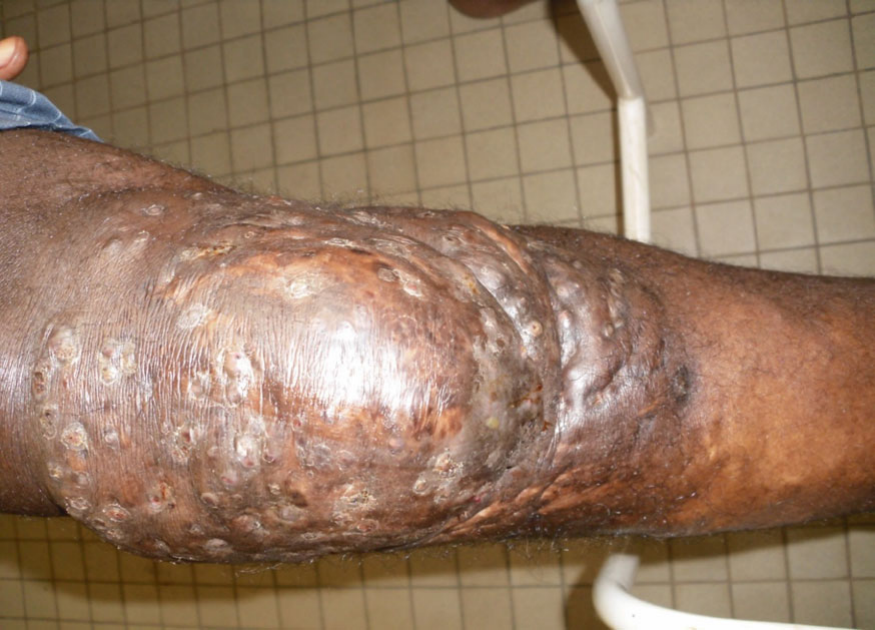
Photography showing the mycetoma triad of mass, multiple discharging sinuses and black grains due to *M. mycetomatis*

**Fig. 2:**
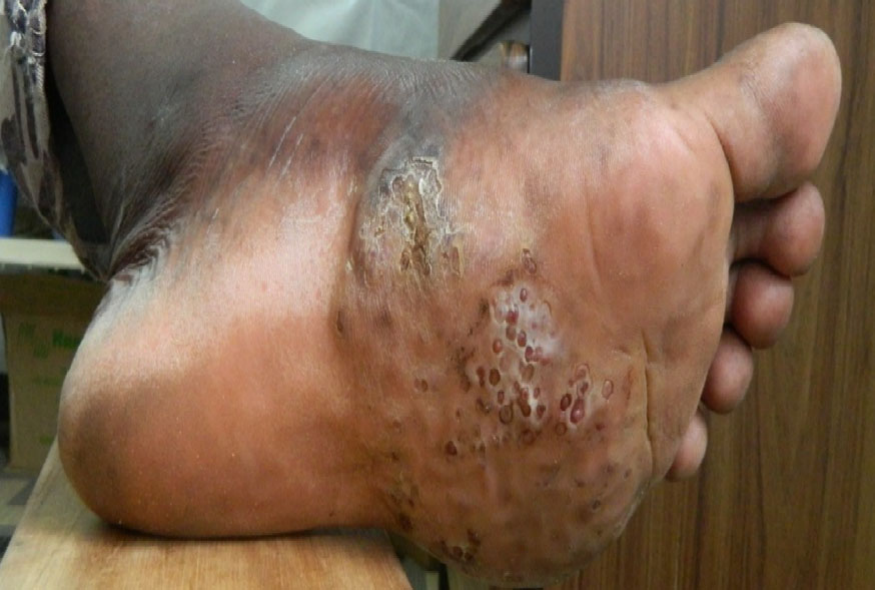
Photography showing the mycetoma of the foot in *A. pelletieri*

Sixty-one applications for mycetoma were recorded with diagnostic confirmation in 33 cases (54.1%). The annual incidence was 1.3 cases. The diagnosis was made in 20 men aged 11–73 years and 13 women aged 9–70 years. The sex ratio (M/F) was 1.5. The average age of patients in our series was 29.7 ± 1.34 years, with extremes of 12 and 73 years. The majority of the patients came from the savanna region (24 of them, 72.7%), including 14 men and 10 women, and other regions of the country (*n* = 9 cases, 27.3%). Depending on the occupation, 69.7% (*n* = 23 cases) were farmers, 18.2% (*n* = 6 cases) traders, and 12.1% (*n* = 4 cases) students. The main sites were the lower limbs (*n* = 21 cases, 54.5%), including 19 cases on the feet, trunk (*n* = 6 cases), scalp (*n* = 4 cases), upper limbs (*n* = 2 cases) (Figs. 1, 2). The epidemiological characteristics of the patients are summarized in Table 1.

**Table 1:** Epidemiological characteristics of patients

Characteristics	Values
*Sex*	
(i) Men	20/33
(ii) Women	13/33
*Age* (*years*)	
(i) Average	29.7.2 ± 1.34
(ii) Extremes	12–73
*Profession*	
(i) Farmers	23/33
(ii) Traders	6/33
(iii) Students	4/33
*Localization*	
(i) Lower limbs	21/33
Feet	19/21
(ii) Trunk	6/33
(iii) Scalp	4/33
(iiii) Upper limbs	2/33

### Histopathological Data

**Fig. 3:**
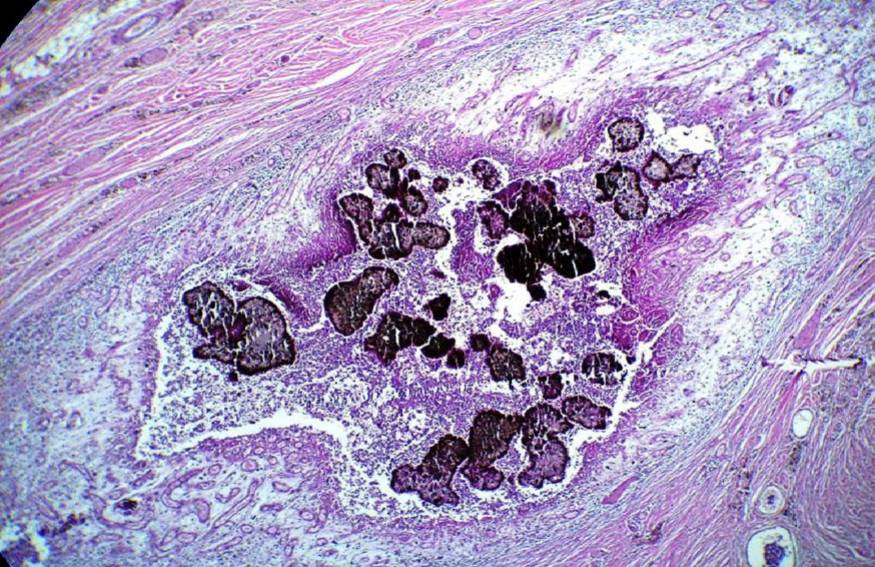
Photomicrograph showing *M. mycetomatis* in tissue section (H and E, × 100)

**Fig. 4:**
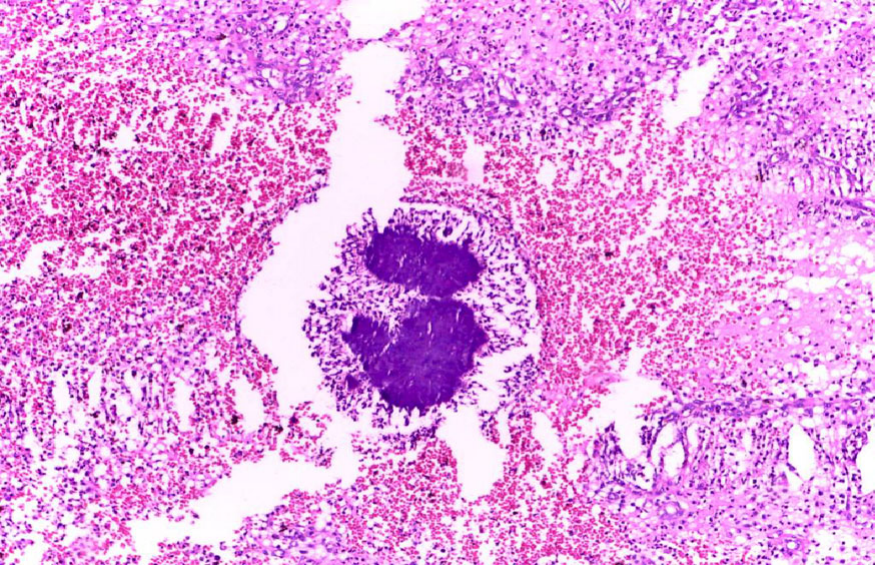
Photomicrograph showing *A. madurae* in tissue section (H and E, × 100)

**Fig. 5:**
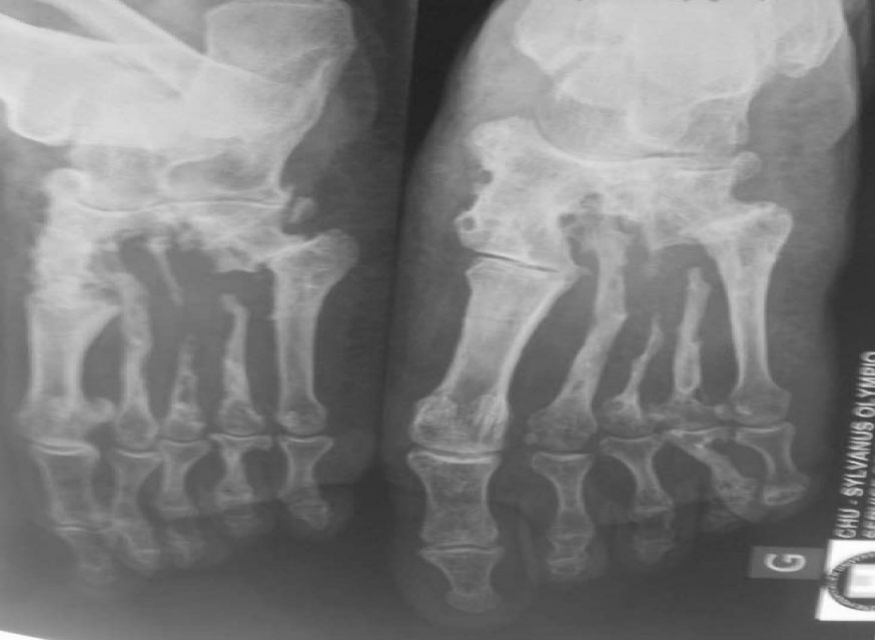
Radiography of feet showing bone destruction

Clinical features were: inflammatory form with fistulae and grains (*n* = 16 cases), tumor (*n* = 10 cases), cystic (*n* = 2 cases) and unspecified (*n* = 5 cases). Diagnosed mycetomas were black (*n* = 24 cases); white grains (*n* = 4 cases); red grains (*n* = 3 cases); yellow grains (*n* = 2 cases). Diagnosed etiologic agents were fungal in 24 (72.7%) and actinomycotic cases in 9 cases (27.3%). Fungal mycetomas were all observed in savannah zones; the fungal agents consisted of *Madurella mycetomatis* (black grains) in 21 cases, *Falcifomispora senegaliensis* (black grains) in 3 cases (Fig. 3). *Actinomadic madurae* (white grains) in 5 cases, *Actinomadurae pelletieri* (red grains) in 3 cases and one case of *Nocardia sp*. (yellow grains) were observed in the other relatively humid regions of the country (Fig. 4). The etiological agents are summarized in Table 2. Six patients had bone lesions such as osteolysis (Fig. 5). They were observed in 4 cases of eumycetomas and two cases of actinomycetomas. Two visceral sites were observed in one patient with *A. pelletieri*; it was localization to the liver, pancreas and right colon.

**Table 2:** Results of pathology according to localization of lesions

	Mn	Fs	Am	Ap	Ns	H	F	Total
Lower limbs	16	2	1	1	1	13	8	21
Trunk	2	1	2	1	0	3	3	6
Scalp	2	0	1	1	0	3	1	4
Upper limbs	1	0	1	0	0	1	1	2
Total	21	3	5	3	1	20	3	33

Mn, *Madurella mycetomatis*; Fs, *Falcifomispora senegaliensis*; Am, *Actinomadura madurae*; Ap, *Actinomadurae pelletieri*; Ns, *Nocardiasp*; F, *female*; M, *male*

## Discussion

Our study shows the rarity of mycetoma because its geographical distribution excludes coastal countries such as Togo. Indeed, mycetomas are endemic on both sides of the 15th parallel north and are observed on a strip of territory from Senegal and Mauritania to the west, to the Republic of Djibouti, Somalia to the East, to Passing through Mali, Niger, Chad, northern Nigeria and Cameroon, and Sudan [11], called mycetoma band. But the number of cases found remains below the reality because of the sub-medicalization; many cases often go unnoticed, either because they do not consult at all or because they are seen in non-specialized health facilities and are not recognized.

We observed a male predominance consistent with data from the literature [1, 2, 9, 11]. Most patients are rural, growers and planters, often walking barefoot or with unsightly sandals. Men and women also work in fields and plantations in rural areas. Women may therefore be considered as more exposed than men. The low proportion of women in our series may suggest a decrease in their reluctance to consult, one of the hypotheses often mentioned to explain the male predominance of the disorder [10, 12].

Feet represent the preferential localization of mycetomas with about 68–82.7% according to studies [13, 14]. The mycetomas can sit on the trunk or buttocks [15]. The scalp or the neck is relatively frequent and mostly poses diagnostic problems [16]. Visceral involvement is rarer, and often secondary to invasion of the mycetoma from a cutaneous focus. Bone and visceral sites determine the functional and vital prognosis of patients with adverse socioeconomic consequences [17].

In our series, five species of etiologic agents are the cause of mycetomas. In the series reported by Pitché et al., these four etiologic agents were responsible for the majority of cases [8, 9]. Philippon et al. in Mauritania, Ndiaye et al. in Senegal identified five etiologic agents [13, 16]. But, the various isolated agents differ from one country to another, as several authors have already pointed out [4, 15]. The etiological distribution of the cases reveals a predominance of forms of fungal origin (eumycetomas) to those of actinomycotic origin (actinomycetomas) [5, 7, 16]. In Africa, black-grained mycetomas are dominated by *M. mycetomatis* and the white grains by *Pseudoallescheria boydii* and the red grain by *Actinomadura pelletieri*; this agrees with the data of our study [7, 13, 17].

Indeed, Togo, which enjoys an annual rainfall ranging between 1200 and 1800 mm depending on the region, is outside the preferred zone of red mycetoma [8]. Our results should lead Togolese practitioners to the introduction of a first-line antibiotherapy in case of actinomycosis that will avoid mutilating surgery. Actinomycotic mycetomas respond well to medical treatment and should under no circumstances be a surgical act [18].

## Conclusion

This report represents a single-center study which provides epidemiologic and histopathological data of mycetoma cases in Togo. Mycetomas are rare diseases in Togo, and our study does not allow us to map the distribution of species in our country. They are dominated by *M. mycetomatis* in fungal forms in forest zone and *Actinomadura madurae* among actinomycoses in savannah zone. With desert advances and population movements, the classical epidemiology of mycetomas in Africa appears to be altered. Thus, every practitioner must know these conditions, because early diagnosis ensures better management and avoids historical complications.
